# Reef Fish Community Biomass and Trophic Structure Changes across Shallow to Upper-Mesophotic Reefs in the Mesoamerican Barrier Reef, Caribbean

**DOI:** 10.1371/journal.pone.0156641

**Published:** 2016-06-22

**Authors:** Dominic A. Andradi-Brown, Erika Gress, Georgina Wright, Dan A. Exton, Alex D. Rogers

**Affiliations:** 1 Department of Zoology, University of Oxford, The Tinbergen Building, South Parks Road, Oxford OX1 3PS, United Kingdom; 2 Operation Wallacea, Wallace House, Old Bolingbroke, Spilsby, Lincolnshire PE23 4EX, United Kingdom; Department of Agriculture and Water Resources, AUSTRALIA

## Abstract

Mesophotic coral ecosystems (MCEs; reefs 30-150m depth) are of increased research interest because of their potential role as depth refuges from many shallow reef threats. Yet few studies have identified patterns in fish species composition and trophic group structure between MCEs and their shallow counterparts. Here we explore reef fish species and biomass distributions across shallow to upper-MCE Caribbean reef gradients (5-40m) around Utila, Honduras, using a diver-operated stereo-video system. Broadly, we found reef fish species richness, abundance and biomass declining with depth. At the trophic group level we identified declines in herbivores (both total and relative community biomass) with depth, mostly driven by declines in parrotfish (Scaridae). Piscivores increased as a proportion of the community with increased depth while, in contrast to previous studies, we found no change in relative planktivorous reef fish biomass across the depth gradient. In addition, we also found evidence of ontogenetic migrations in the blue tang (*Acanthurus coeruleus*), striped parrotfish (*Scarus iserti*), blue chromis (*Chromis cyanea*), creole wrasse (*Clepticus parrae*), bluehead wrasse (*Thalassoma bifasciatum*) and yellowtail snapper (*Ocyurus chrysurus)*, with a higher proportion of larger individuals at mesophotic and near-mesophotic depths than on shallow reefs. Our results highlight the importance of using biomass measures when considering fish community changes across depth gradients, with biomass generating different results to simple abundance counts.

## Introduction

Mesophotic coral ecosystems (MCEs; zooxanthellate coral reefs from 30m to approximately 150m depth) are understudied [1], yet may have a linear extent as great as shallow coral ecosystems [2]. Mapping of MCEs has led to suggestions that identifying their full distribution may double overall reef area at regional scales [3]. Much recent interest in MCEs has focused on whether they act as refuges for threatened shallow-reef species [4]. Many threats faced by reefs, such as bleaching, direct storm damage, local pollution and overfishing have previously been assumed to decline in severity with depth [5]. However, this proposed importance as a depth refuge has recently been challenged, as it has become clear that MCEs face many of these threats as well [6].

Zooxanthellate coral reefs in tropical and subtropical areas can broadly be divided into three depth zones: i) shallow coral reefs (0-30m), ii) the upper mesophotic zone (30-60m) and iii) the lower mesophotic zone (60m-maximum local depth of zooxanthellate corals, typically between 150 and 200m) [7,8]. MCEs are often found in association with shallow coral reefs, as reefs extend down the depth gradient from shallow to mesophotic depths. Armstrong and Singh [9] observed that temperature and light availability are important for MCE communities to establish, but sediment channelling at depth separated highly developed MCEs from poorly established communities at very close proximity.

Reef fish and coral distributions from shallow reefs to MCEs exhibit similar patterns in species richness, abundance and biomass, although there is geographical variation in exact transition depths between communities. For example, in the Caribbean, over 75% of shallow coral species extend into MCEs [5]. Bejarano *et al*. [10] compared fish populations on MCEs to 70m and found approximately 75% of fish species on Puerto Rican MCEs could also be found on shallower reefs, although many of these did not occur past 60m. Research in the Marshall Islands reported a shallow fore reef fish community that extended from the surface to approximately 75-90m, after which many shallow species were lost [11]. In the Red Sea, shallower fish species were found to drop off rapidly below 30m [12]. Many of these differences in transition points between shallow and mesophotic fish communities are likely to be driven by local and regional variations in environmental conditions, although the general trend of the upper mesophotic zone harbouring shallow fish species appears widespread.

Trophic groups also respond differently to depth across the shallow to mesophotic gradient. Generally reef fish communities shift from herbivore dominance at shallow depths to carnivore dominance (mainly zooplanktivores) at mesophotic depths [7]. This trend has been observed in the Caribbean [10,13], Brazil [14,15], the Marshall Islands [11] and the Red Sea [12,16]. Regionally in the Caribbean, planktivores account for 22% of fish species recorded on MCEs [7]. In the Gulf of Mexico Weaver *et al*. [17] found fish from the subfamily Anthiinae (small planktivores in the family Serranidae) were so abundant on MCEs that they considered them keystone species, transferring energy from the water column to the reef.

At a local scale, the structural complexity provided by corals and other benthic species is critical for MCE fish communities to thrive. For example, large grouper abundance in Puerto Rico has been observed to peak at 25m depth, but only in association with *Montastrea spp*., the dominant scleractinian coral at those depths [13]. Other studies have reported the grouper *Mycteroperca phenax* associated with high structural complexity reefs at 70-100m depth in Puerto Rico [18]. In the Red Sea, turnover in fish community composition at mesophotic depths appears highly correlated with a reduction in branching coral abundance [12]. Other fish distributions are directly restricted based on benthic associations. Anemonefish on MCEs, both in the Red Sea [19] and the Great Barrier Reef [20], are likely to be driven by the distribution of their host anemones rather than reaching any physiological depth limit for the fish themselves.

Many fish species incorporate depth into their life histories, migrating bathymetrically as they mature or for spawning [21]. These ontogenetic migrations are driven by the trade-off between maximising food availability and minimising predation risk [22]. Ontogenetic migrations have been observed in many Caribbean fish species with young juveniles typically found in mangroves, seagrass beds and back reefs, before migrating to reef crests and slopes as they mature [23]. However, depth distributions in species life histories are far from consistent. In the Gulf of Aqaba, Red Sea, juvenile zebra angelfish (*Genicanthus caudovittatus*) abundance peaks at 60-65m, where 80% of juveniles were found, compared to adult peak abundance at 30m [24].

Within the Caribbean and western Atlantic, existing research is generally limited to a few locations such as Puerto Rico [10,13], Brazil [14,15,25], Curaçao [26], Bermuda [26] and the Gulf of Mexico [17], and has mostly focused on comparing visual in-water or video counts of species abundance. Using fish biomass provides a better understanding of ecosystem functions across the depth gradient, and the dominant trophic groups driving these patterns. Switching community analysis from abundance to biomass could significantly affect our understanding of tropic structuring as, for example, piscivorous fish tend to be larger bodied than planktivorous or herbivorous reef fish [27], so simple abundance comparisons are unlikely to determine the relative ecological functions performed by different groups. Length information also allows patterns in fish life history to be identified, including ontogenetic migrations across the depth gradient. In this study we investigate how reef fish communities change across a shallow to upper mesophotic reef gradient on the southern Mesoamerican Barrier Reef by addressing both abundance and biomass. We use a diver-operated stereo-video system to enable accurate reef fish biomass estimates to be made along a Caribbean shallow-MCE depth gradient for the first time. Specifically we address changes in dominant fish trophic groups, to test whether previously reported patterns elsewhere on MCEs occur in the southern Mesoamerican Barrier Reef, and the use of the biomass metric rather than abundance affects interpretation of results.

## Materials and Methods

### Data collection

**Fig 1:**
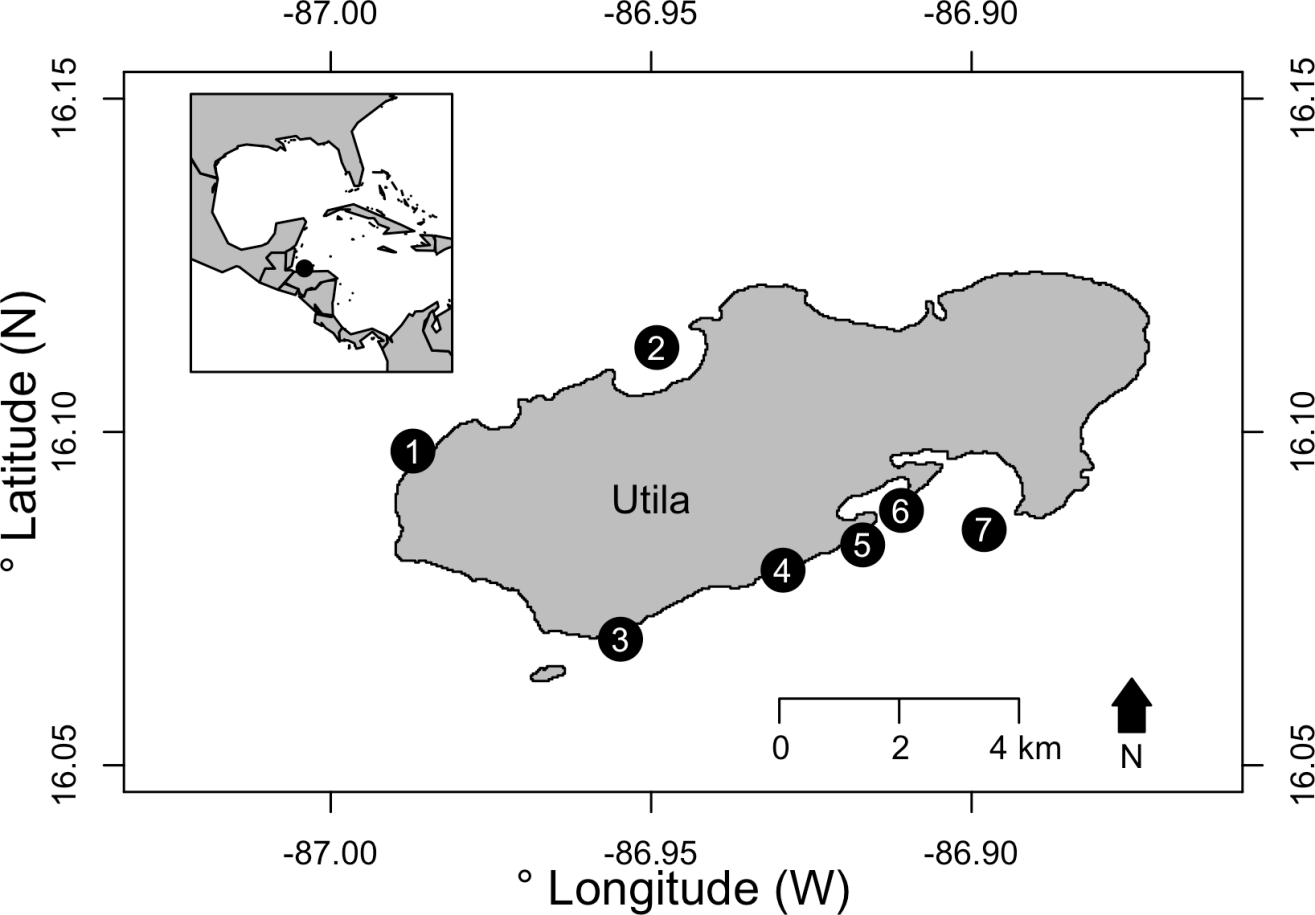
Map of study sites around Utila, Honduras. Survey locations are marked, with numbers indicating sites as follows: (1) Spotted Bay, (2) The Maze, (3) Stingray Point, (4) Little Bight, (5) Black Coral Wall, (6) Coral View and (7) Lighthouse Reef. Inset map shows the location of Utila relative to the Caribbean region. Map sourced from GADM database of Global Administrative Areas under a CC BY licence with permission.

Surveys were conducted at seven fringing coral reef sites around Utila, Bay Islands, Honduras (see S1 Table for GPS locations). Utila is located at the southern end of the Mesoamerican Barrier Reef. Two sites (The Maze and Spotted Bay) were located on the island’s exposed north shore, and five were located on the more sheltered south shore (Stingray Point, Little Bight, Black Coral Wall, Coral View and Lighthouse Reef) (Fig 1). On the south shore the reef slope reaches a maximum depth of 40-60m where the seabed levels off stretching to mainland Honduras. South shore MCEs tend to be extensive patch reef systems, with large areas of reef separated by areas of sand. On the north shore of Utila, the reef slope continues to >100m. Shallow reefs at south shore sites consist of a spur and groove system, whereas shallow reefs on the north shore are on steeper reef walls. Field Permits were issued by the Instituto de Conservacion Forestal (ICF), Honduras.

Transects were conducted using a diver-operated stereo-video system (SVS; SeaGIS, Melbourne, Australia), composed of two Canon HFS21 high definition video cameras (see following for system overview: [28–30]). SVS allows fish communities to be recorded more quickly in water than traditional underwater visual census (UVC) technique, and for fish biomass to be estimated more accurately [31]. At each site, six transects were conducted at 5m and 15m and four transects were conducted at 25m and 40m. Fewer transects were conducted at 25m and 40m depths because of increased logistical challenges associated with depth. However, using an unbalanced study design such as this increases overall statistical power to discern ecological changes across the depth gradient [32].

Transects were 50m long following the reef contour, with a 10m interval between replicates, and were surveyed by an SVS operator and a second diver responsible for distance measurement. To minimise potential disturbance to the fish community while cameras were set to record and synchronised, transects were begun with the cameras pointing vertically down, and at 10m the SVS operator was alerted via a fin tug to indicate the start of the transect proper. At this point cameras were pointed along the reef, with another fin tug indicating the transect end after a further 50m. Cameras were angled at approximately 20 degrees downwards, and kept approximately 0.5m above the seabed, filming the reef scape along the transect. Transects took approximately 4 minutes to film with divers using open circuit accelerated decompression SCUBA diving.

Footage was analysed in EventMeasure software v3.51 (SeaGIS, Melbourne, Australia) allowing the calibrated SVS footage to be synchronised and fish total lengths to be measured. EventMeasure also resolved centre points of each individual fish encountered into distances on a three-dimensional coordinate system, allowing us to exclude fish outside 2.5m either side and 5m in front of the camera system. Side distance restriction maintains a consistent belt along the transect, while front distance restriction prevents variations in visibility (e.g. turbidity, light intensity) from influencing data.

After completing the fish surveys, benthic videos were filmed by the SVS operator as the survey team returned along each 50m transect using a Cannon HFS21 or GoPro Hero 3 Silver video camera. This allowed the SVS operator to swim slowly along the transect with the camera pointing vertically down and held approximately 0.3m above the benthos, keeping the transect tape within the frame at all times. Point-intercept analysis was conducted, recording the benthic substrate at 0.25m intervals following English *et al*. [33].

SVS and benthic video analyses were conducted by a volunteer team of undergraduate students. Volunteers were extensively trained prior to participation, having specifically (i) completed a week long Caribbean coral reef ecology course combining lectures with in-water practicals where experienced researcher pointed out fish species for them to observe, (ii) passed Caribbean coral, macroalgae and reef fish species identification written exams (pass grade 80%) and had any incorrectly identified reef fish discussed with them, (iii) completed a training tutorial in EventMeasure analysis, and (iv) conducted 12 training stereo-video transect analyses in EventMeasure where an fish identification expert reviewed each video on completion allowing commonly mistaken fish species to be highlighted to the volunteers. While conducting the actual transect video analysis, volunteers had a copy of Humann and Deloach [34] and were able to discuss or confirm any fish they were unsure of with other volunteers or an experienced researcher. All fish were identified to species level following Humann and Deloach [34]. A detailed description of the raw data is contained in S1 Appendix and the raw data files available in S1, S2 and S3 Data.

### Analysis

Fish length measurements were converted into biomass using Eq 1.

$$W=aL^{b}$$ (1)

Where *L* represents the fish length in centimetres, *W* the weight in grams and *a* and *b* published species-specific conversion constants from Fishbase (accessed September 2014). Where conversion constants were not available, the genus mean was used. Where fish length measurements were not possible (e.g. because of the angle of an individual to the cameras), the individual was recorded and the mean length for that species at that depth and site applied or, where no site and depth con-specifics were available, the mean across all depths and sites. These fish were excluded from all analysis of median fish lengths. Variation in coverage of key benthic groups (hard coral, soft coral, macroalgae, sponge and sand) were calculated as percentage cover. Fish species richness, abundance and biomass were calculated as mean per transect (250m^2^).

To compare benthic habitats between north and south shores, sites and depths, a non-parametric permutational analysis of variance (PERMANOVA) was used [35]. For fish biomass, a Bray-Curtis dissimilarity matrix was constructed based on fourth root transformed fish species biomass for both non-metric multidimensional scaling (nMDS) and PERMANOVA. MDS plots were constructed to illustrate the relationship between fish communities and the availability of hard substrata using the vegan package [36] in R [37]. For comparisons between fish communities we included percentage hard substratum as a variable in the PERMANOVA model. Hard substratum was defined as benthic cover that was not mud, sand or rubble following Gratwicke and Speight [38]. Fish community permutations were constrained by site. All PERMANOVAs were run for 99999 permutations using the ‘adonis’ function in vegan. Because of the multifactor and unbalanced nature of our data, when testing interactions sums of squares for model terms are non-independent [35]. To address this ‘adonis’ uses Type I (sequential) sums of squares, with each term sequentially fitted after taking account of the previously fitted terms. This approach is considered appropriate for nested experimental designs where terms naturally exhibit a hierarchical ordering [35].

To investigate variation in the proportion of fish feeding guilds we used the Caribbean fish feeding guild classification by Micheli *et al* [39] to allocate each species to a trophic group. Trophic groups were: herbivores, planktivores, piscivores, omnivores, invertebrate feeders and fish species that feed as both piscivores and invertebrate feeders, which we call carnivores. We calculated the proportion of the overall fish community each trophic group composed at each depth. We used a univariate PERMANOVA based on Euclidean distances and constrained by site to test for differences between the total biomass at 5m and 40m and the proportion of biomass at 5m and 40m each trophic group comprised. Only 5m and 40m data were used to allow a simple comparison between the fish community on shallow reefs at the top of the reef slope, with those found deeper in the upper-mesophotic zone. Proportions were calculated by transect to allow permutations within sites, with summary mean proportions calculated by averaging across sites. To test whether changes in trophic groups along the depth gradient were caused by species gains, losses or turnover we conducted separate Principal Components Analysis (PCA) for each trophic group using the fourth root transformed multivariate species biomass data [40]. Pearson’s correlation coefficients between each species’ biomass in the trophic group and the first PCA axis for that trophic group were calculated. For species that exhibited a correlation coefficient |*r*|≥ 0.3 we further tested whether the species biomass or proportion of trophic group biomass changed between 5m and 40m with a univariate PERMANOVA using Euclidean distances.

Kernel density estimates (KDEs) were used for fish length distribution visualisation at each depth and to test differences in the body length distributions for species identified as correlating with the PCA analysis. Bandwidths for the KDEs were chosen by the Sheather-Jones selection procedure [41] using the ‘dpik’ function in the ‘KernSmooth’ package [42], following Langlois *et al* [43]. KDEs are used when the relatedness between samples is not known and the data are not normally distributed. Differences in length distributions were tested by a permutation test (*n* = 10,000) using the function ‘sm.density.compare’ in the R package ‘sm’ [44]. This uses permutation to test for differences in area between the two probability density distributions.

In order to have enough replicate length measurements from individual species to allow identification of whether they exhibited differing body size with depth, we grouped all lengths across all sites and from 5m and 15m as shallow and from 25m and 40m as deep. For the resulting seven species with 20 or greater individuals measured in both shallow and deep groups we calculated KDEs.

## Results

### Fish species richness, abundance and biomass

**Fig 2:**
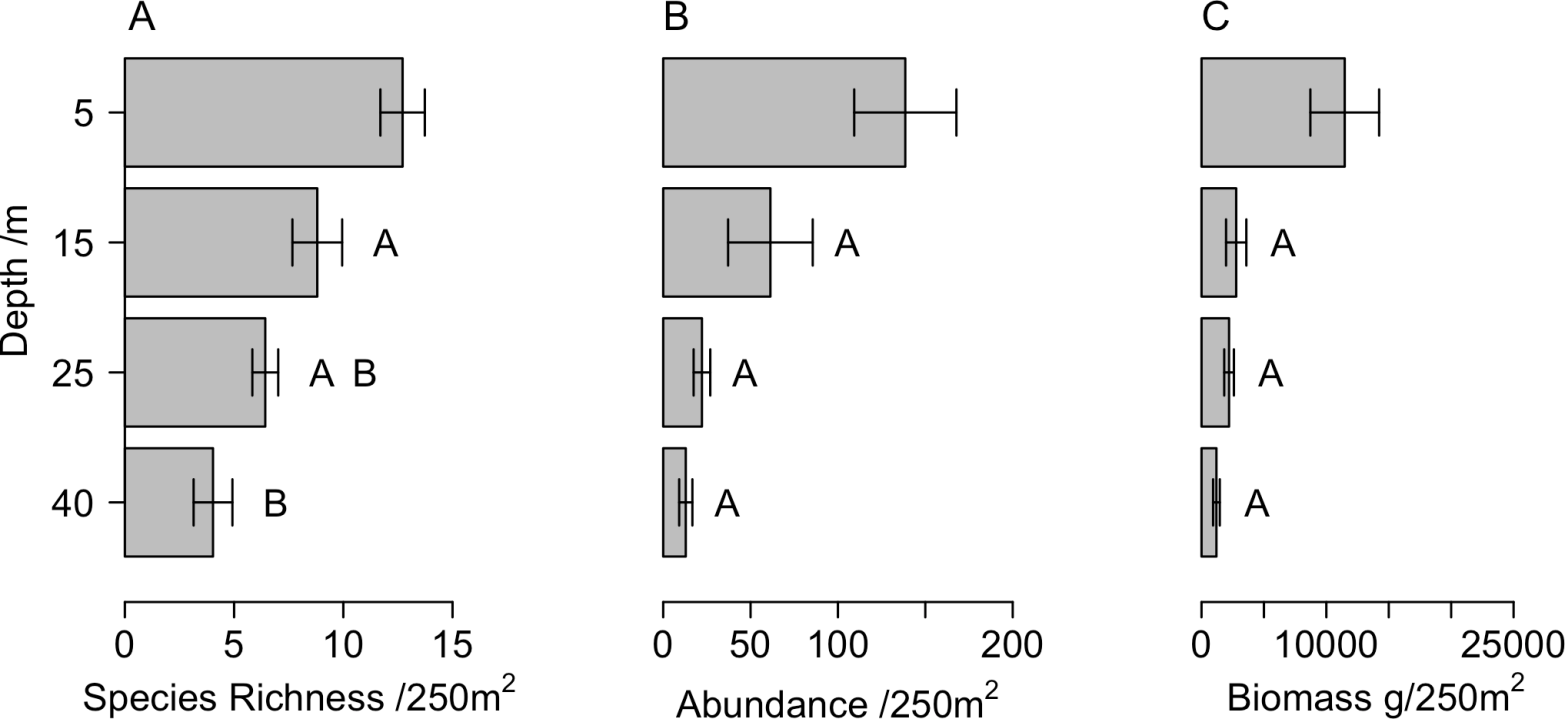
Changes in fish communities with depth. (A) Species richness, (B) abundance, (C) biomass down the depth gradient. Figure shows mean and SE. Letters indicate statistically different groups at the p<0.05 level.

**Fig 3:**
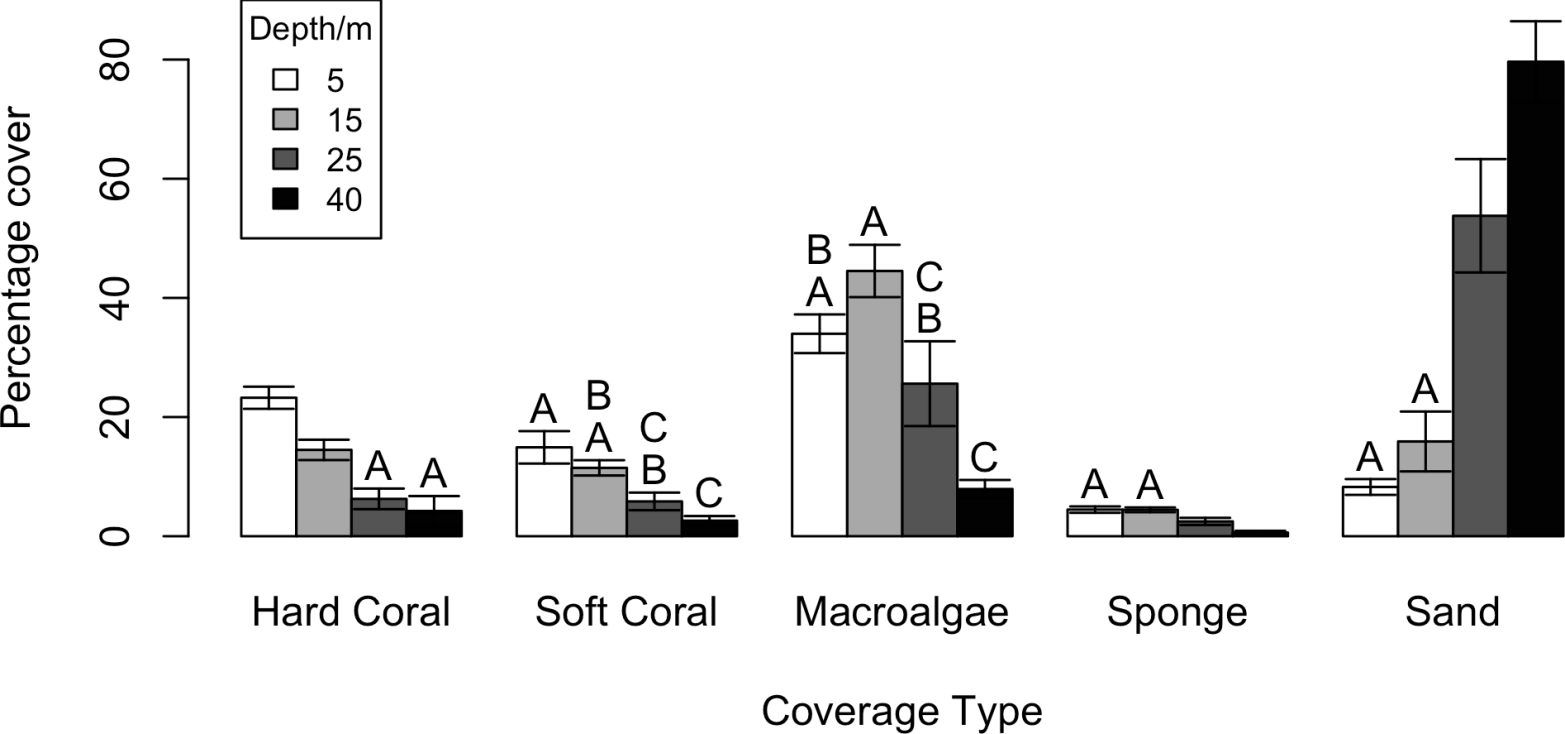
Change in percentage cover of key benthic groups with depth. Figure shows mean and SE, letters indicate differences (p>0.05 with one-way ANOVA).

**Fig 4:**
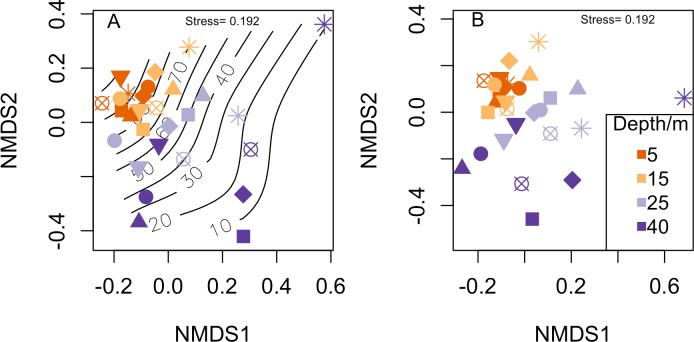
Non-metric multidimensional scaling plot of fish biomass weighted communities for each depth and site. (A) Overall NMDS plot showing community clustering by depth, contour lines indicate percentage cover of hard coral and (B) fish community biomass standardised by percentage hard coral availability at each site.

Fish species richness gradually declined with depth from an average per transect of 12.7 ± 1.0 (mean ± standard error) species at 5m to 4.0 ± 0.9 species at 40m (Fig 2A), whereas abundance and biomass rapidly declined between 5m and 15m and then did not change with further increases in depth (Fig 2B & 2C). Hard substratum availability declined with depth from 92% at 5m, to 20% at 40m. Many hard substratum-associated benthic groups such as hard corals, soft corals and sponge were found to decline across the depth gradient (Fig 3). Macroalgae coverage shows a less clear pattern at shallower depths, but is lower at mesophotic than shallow reef depths (Fig 3). When visualising fish communities at each site and depth using nMDS it becomes apparent that the fish communities are highly correlated with the availability of hard substratum (Fig 4A), yet when standardised on hard substratum availability fish communities still group on depth (Fig 4B), suggesting differences in hard substratum availability with depth was not the only driver of fish community structure. Hard substratum availability explained a significant amount of the variation along with individual sites (Table 1). Hard substratum effects varied with shore, with higher hard substrate at MCEs at the Maze on the north shore (59.8 ± 14.19%) compared to Spotted Bay and the south shore sites (13.8 ± 2.0%). Depth was also significant in explaining fish communities. Particularly interesting is both the site*depth and shore*depth interactions, implying that while sites had different patterns in fish community with depths, the two north shore sites’ fish communities vary with depth differently to those on the south shore. Three-way interactions were tested when the model was run, but were found not to be significant.

**Table 1:** Testing the effects of hard substrata, shore, site and depth on the recorded fish community using PERMANOVA.

	df	SS	MS	Pseudo-F	p(perm)
Hard substrata	1	2.304	2.30399	6.6586	<0.001
Shore	1	1.028	1.02797	2.9709	0.344
Site	5	2.205	0.44094	1.2743	<0.001
Depth	3	2.629	0.87641	2.5329	<0.001
Hard substrate*Shore	1	0.557	0.55716	1.6102	0.013
Hard substrate*Site	5	2.460	0.49191	1.4216	0.001
Hard substrate*Depth	3	1.376	0.45881	1.3260	0.014
Shore*Depth	3	1.564	0.52144	1.5070	<0.001
Site*Depth	15	6.450	0.43003	1.2428	<0.001
Hard substrate*Shore*Depth	3	1.179	0.39309	1.1360	0.128
Hard substrate*Site*Depth	15	5.806	0.38707	1.1187	0.071
Residuals	79	27.335	0.34602		
Total	134	54.894			

Analysis conducted on a Bray-Curtis dissimilarity matrix based on fourth-root transformed fish biomass data. Permutations were run 9999 times and constrained by site.

### Fish trophic structuring

**Fig 5:**
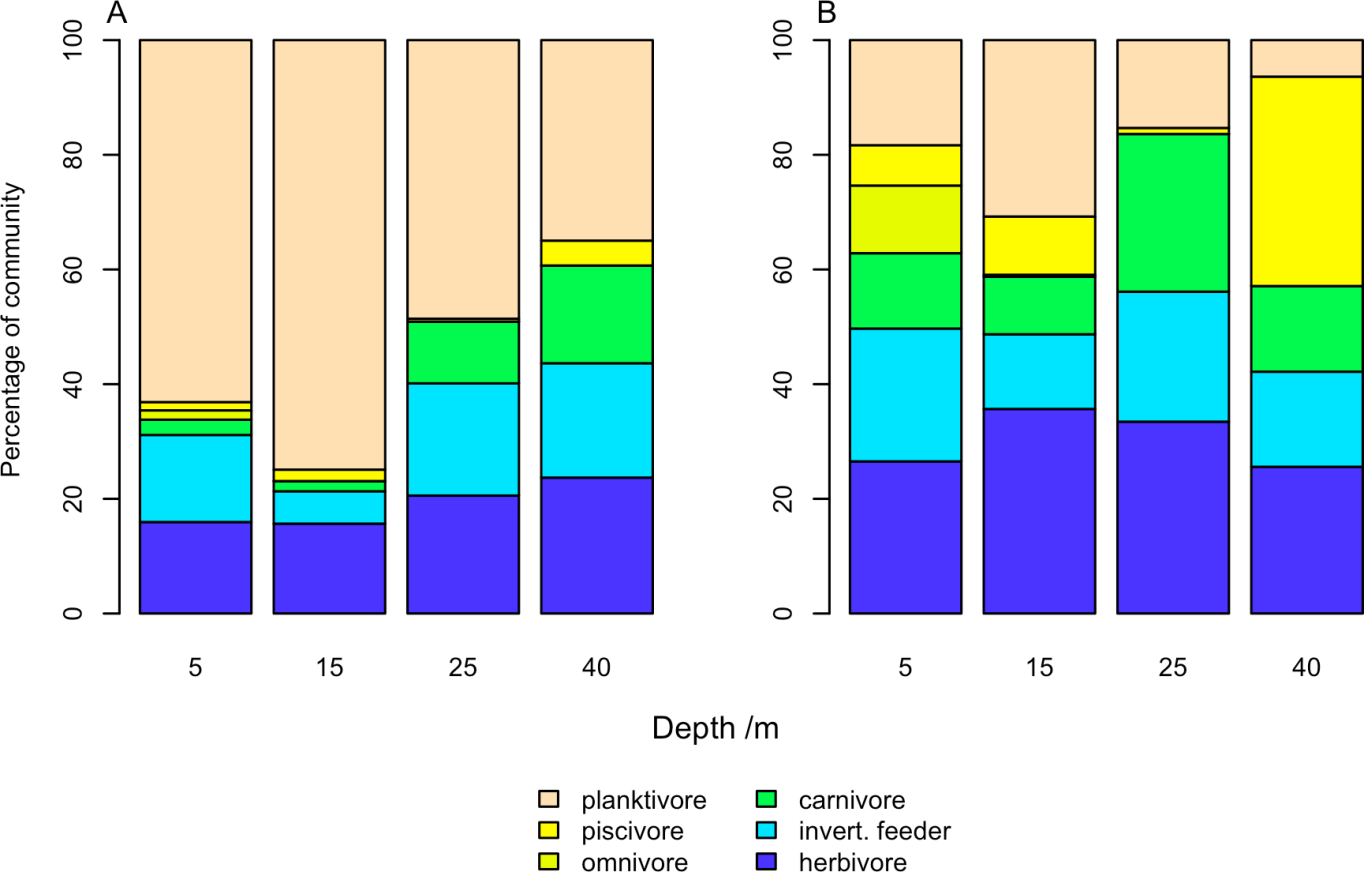
Change in percentage of different trophic groups with depth. Fish community was weighed by (A) abundance and (B) biomass. Percentage abundance and biomass of each trophic group was calculated by summing all fish identified on all transects at all sites in a depth band.

Grouping fish into trophic groups allowed changes in dominant feeding strategies with depth to be investigated. Within all trophic groups fewer species were observed at 40m than at 5m (Table 2). Invertebrate feeders had the greatest species richness at both 5m and 40m, with 25 and 17 species respectively, followed by herbivores with 17 and 12 species. Grouping communities based on abundance (Fig 5A) or biomass (Fig 5B) was found to have major effects on the dominant trophic groups recorded. Planktivorous fish were found to be the dominant trophic group at all depths when calculating community proportion based on abundance, representing 63% of all fish recorded at 5m (3619 individuals recorded in total across all transects) and 35% of fish recorded at 40m (121 individuals recorded). When using biomass weighted communities, however, the planktivores composed a much smaller percentage of the community, only 18% at 5m and 6% at 40m, and the biomass was more evenly distributed between trophic groups (Fig 5). At shallow depths in the biomass-weighted community herbivores were the largest group, making up 27% of the community at 5m, whereas on MCEs piscivores became the largest trophic group at 37% of the community (Fig 5). Fish biomass was significantly lower at 40m than 5m for herbivores, invertebrate feeders and planktivores, while carnivores, omnivores and piscivores has similar biomass between 5m and 40m (Table 2). Considering each trophic group biomass as a percentage of the total biomass recorded per transect at each depth, we found only herbivores had a decline in proportion of the community (38.97% ± 5.78 at 5m to 18.96% ± 2.96 at 40m), while piscivores increased their proportion of the community (6.44 ± 1.86 at 5m to 20.14 ± 8.62 at 40m) (Table 2).

**Table 2:** Comparison of fish trophic groups between 5m and 40m depths across all sites.

Trophic Group	Species Richness		Mean Biomass per transect/g ± SE				Mean Percentage Biomass per transect ± SE			
	5m	40m	5m	40m	Test statistic	p(perm)	5m	40m	Test statistic	p(perm)
Herbivore	17	12	2972.95 ± 724.14	300.73 ± 112.14	8.24	<0.001	38.97 ± 5.78	18.96 ± 2.96	9.06	0.004
Invertebrate feeder	25	17	2594.35 ± 1318.35	194.74 ± 51.60	5.10	0.003	19.60 ± 5.13	22.42 ± 8.46	0.21	0.618
Carnviore	6	3	1471.81 ± 658.08	175.67 ± 117.27	2.00	0.114	12.92 ± 2.43	9.59 ± 5.91	0.40	0.540
Omnivore	1	0	3085.71 ± 1261.70	0.00 ± 0.00	1.66	0.280	7.92 ± 0.97	0.00 ± 0.00	2.56	0.182
Piscivore	6	4	788.34 ± 393.47	429.07 ± 250.11	0.38	0.611	6.44 ± 1.86	20.14 ± 8.62	4.58	0.034
Planktivore	6	4	2053.30 ± 792.09	74.83 ± 56.06	4.66	0.001	18.67 ± 2.56	14.61 ± 5.26	0.50	0.503

Species richness is the total number of unique species observed at all transects at all sites in the respective depth band. Mean biomass and mean percentage biomass are given per transects, along with the results of univariate PERMANOVAs constrained by site to test for differences with depth.

Within each trophic group we identified which species were most likely to explain the patterns observed by the trophic group as a whole based on correlations with a PCA (Table 3, S2 Table). Within herbivores we found *Acanthurus* spp. did not change in total biomass or the proportion of herbivore biomass they composed between 5m and 40m. All parrotfish (genus *Scarus* and *Sparisoma*) declined in biomass with depth, with four species (*Sparisoma aurofrenatum*, *Sparisoma chrysopterum*, *Sparisoma rubripinne* and *Sparisoma viride*) also declining as a proportion of the herbivore community. The two damselfish species (*Microspathodon chrysurus* and *Stegastes adustus*) declined in biomass with depth, but did not change in the proportion of the herbivore community they made up. This suggests the decline in herbivores observed between 5m and 40m is caused both by a loss of species with depth, and a decline in biomass as a proportion of the herbivore community at 40m. In piscivores, the only trophic group to increase as a proportion of the community between 5m and 40m, there were no changes in mean biomass or percentage of piscivore biomass made up by any of the three species correlating with the PCA (*Aulostomus maculatus*, *Caranx ruber* and *Sphyraena barracuda*). Within the planktivores we identified two species (*Clepticus parrae* and *Chromis cyanea*) that significantly declined as a proportion of total community biomass, suggesting the changes observed in proportion of planktivores observed with depth was also driven by species loss.

**Table 3:** Species correlating with changes in their trophic group with depth.

Trophic Group		Mean Biomass per transect			Mean Percentage of Trophic Group Biomass per transect		
		Pseudo F	p(perm)	Direction	Pseudo F	p(perm)	Direction
Herbivores							
	*Acanthurus bahianus*	2.87	0.051	-	0.67	0.405	-
	*Acanthurus coeruleus*	1.66	0.178	-	1.12	0.306	-
	*Microspathodon chrysurus*	7.93	0.002	↓	0.06	0.874	-
	*Stegastes adustus*	18.22	<0.001	↓	0.10	0.786	-
	*Scarus iserti*	5.01	0.010	↓	0.32	0.580	-
	*Sparisoma aurofrenatum*	3.63	0.012	↓	5.12	0.012	↓
	*Sparisoma chrysopterum*	6.13	0.006	↓	4.77	0.011	↓
	*Sparisoma rubripinne*	6.05	0.009	↓	4.67	0.023	↓
	*Sparisoma viride*	18.19	<0.001	↓	12.61	0.001	↓
Invertebrate Feeders							
	*Abudefduf saxatilis*	4.23	0.012	↓	33.27	<0.001	↓
	*Lutjanus apodus*	2.47	0.073	-	6.77	0.010	↓
	*Lutjanus jocu*	0.63	0.904	-	0.00	1.000	-
	*Lutjanus mahogoni*	2.20	0.053	-	9.84	0.003	↓
Omnivores							
	*Kyphosus sectatrix*	1.59	0.280	-	2.86	0.142	-
Piscivores							
	*Aulostomus maculatus*	1.91	0.253	-	2.09	0.252	-
	*Caranx ruber*	0.82	0.567	-	0.00	1.000	-
	*Sphyraena barracuda*	0.89	0.471	-	0.91	0.568	-
Planktivores							
	*Clepticus parrae*	0.93	0.401	-	6.96	0.008	↓
	*Chromis cyanea*	5.60	0.001	↓	25.99	<0.001	↓

Results for species exhibiting a >0.3 absolute value correlation with the first Principle Components Analysis axis for their trophic group. Correlations were tested using a PERMANOVA based on biomass per transect, and the percentage of biomass contribution to total biomass of that guild per transect. Significance of changes are indicated and the direction of change shown. See S2 Table for expanded version of this table.

### Fish length distributions

**Fig 6:**
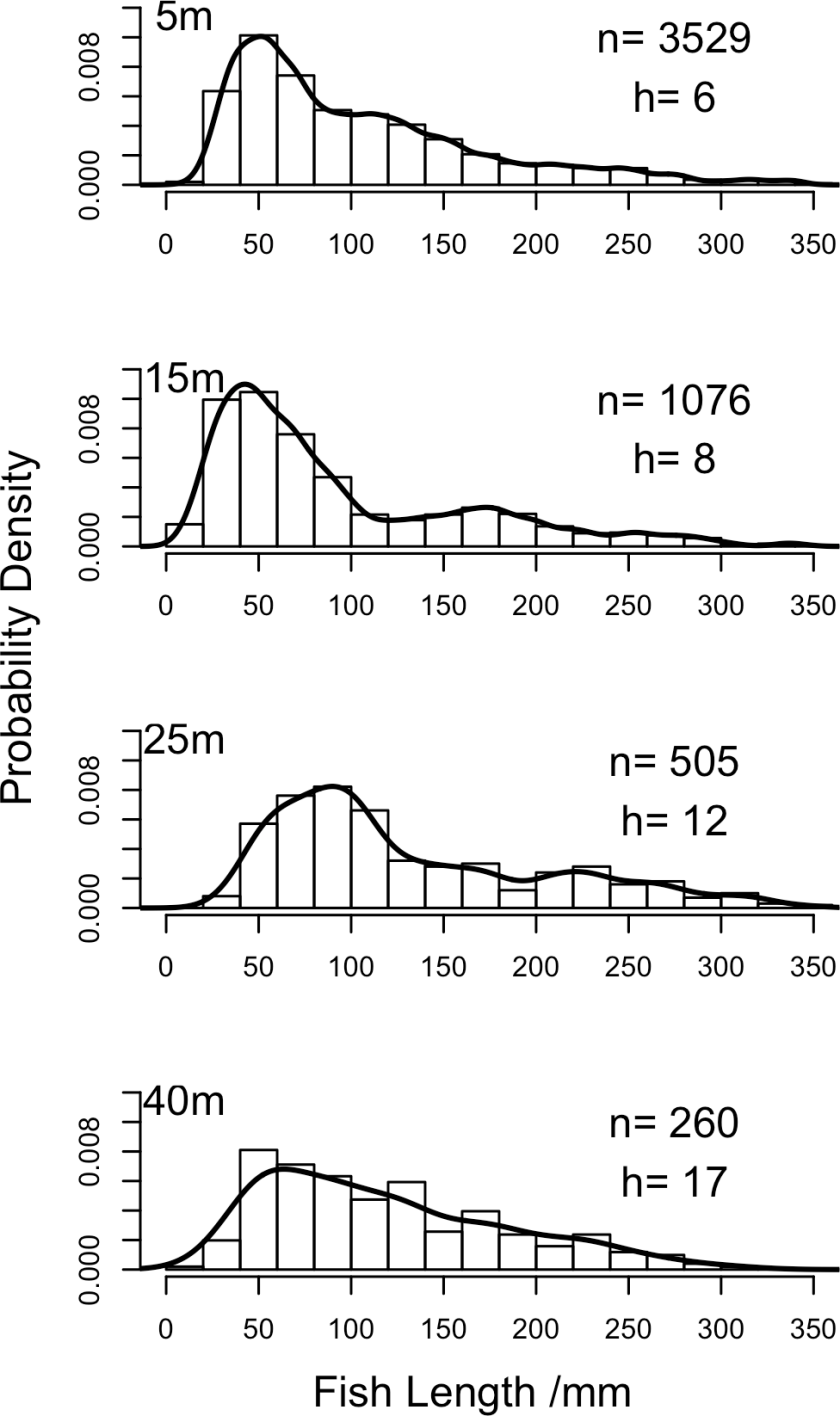
Whole fish community length distributions at each depth across all transects and sites. Number of fish lengths recorded (n), and the separation bandwidths to produce kernel density estimates (h) calculated by Sheather-Jones selection procedure are shown.

**Fig 7:**
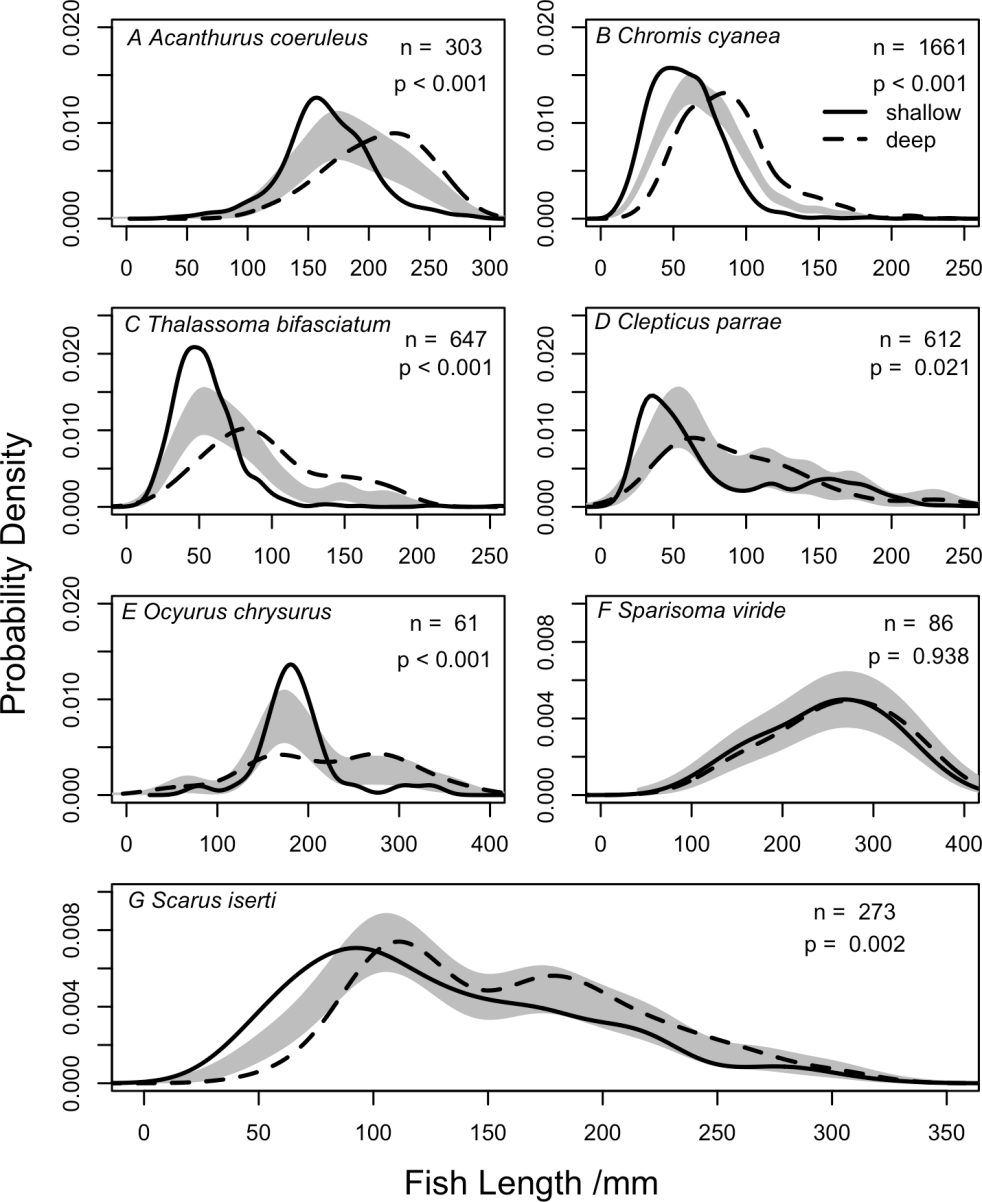
Comparison of kernel density estimates for four fish species between shallow and deep reefs. Comparisons based on all fish individuals measured on shallow (5 and 15m) and deep reefs (25 and 40m) at all sites. Grey shaded regions represent one standard error either side of the null model, n = number of individual fish measured, p indicates whether the length distributions are significantly different based on permutation tests.

To better understand the differences between abundance- and biomass-weighted fish communities (Fig 5) we plotted total lengths for all fish at each depth (Fig 6), finding that at shallow depths a larger proportion of the community comprises smaller reef fish. When grouping the 5m and 15m data together as shallow and the 25m and 40m data as deep across all sites only seven species were measured more than 20 times at both shallow and deep depths. These species were: the herbivores the blue tang, (*Acanthurus coeruleus*), the stoplight parrotfish, (*Sparisoma viride*) and the striped parrotfish (*Scarus iserti*), three planktivores, the blue chromis (*Chromis cyanea*), the creole wrasse (*Clepticus parrae*), and blueheaded wrasse (*Thalassoma bifasciatum*) and a carnivore, the yellowtail snapper (*Ocyurus chrysurus*). Six of these seven species exhibited significant differences in their fish length structure between shallow and deep locations, with larger individuals found deeper when tested using a permutational test of distribution (Fig 7). The stoplight parrotfish was the only species that did not exhibit a change in length distribution with depth (Fig 7F). The striped parrotfish exhibited a bimodal length distribution at deeper depths (Fig 7G). We tested whether this was caused by combining the length distributions from 25m and 40m but found no difference between fish recorded at these two depths (permutational test of equality, *n* = 75, *p* = 0.76).

## Discussion

### Fish species richness, abundance and biomass changes across the depth gradient

Our results showed a continuous decline in fish species richness with increased depth, which contrasts with findings from many other Caribbean locations. For example across a 15-50m depth gradient in Puerto Rico the greatest fish species richness was recorded at 25m [13], whereas off North Carolina this peak in species richness occurred deeper in the 52-98m range [45]. Outside of the western-Atlantic, in the Red Sea species richness declined from shallow to mesophotic reefs, but with a small peak at 30m; the boundary between the shallow and mesophotic, caused by a mix of deep and shallow specialist and depth generalist species [12]. In Hawaii the greatest richness was observed in the 31-60m range, though in this study it was likely the 0-30m range was under sampled [46].

The differences between our findings and those of previous work may be explained by the maximum extent of the reefs at our study sites. The south shore of Utila has a maximum depth range of 40-60m, with low hard substratum availability at 25m and 40m. This suggests that observed fish communities at these sites represent the shallow-water community in the absence of fully developed deeper MCEs, reducing the number of deep specialist fish species present. Reefs at our two north shore sites extend to >100m and we did see a significant shore*depth interaction (Table 1). This suggests a difference in the turnover of fish communities with depth at sites on the north shore of Utila compared to the south shore. Many other factors could also in part explain this interaction; the north shore sites have larger shallow reef communities by area, with extensive back reefs behind the reef crest, and greater wave exposure for much of the year than the south shore sites, and so shallow reef differences could be an important driver. Coastal development has also focused on the south shore of Utila, resulting in the south shore reefs facing increased sedimentation from mangrove forest clearance and south shore lagoon dredging.

Declines in abundance of fish with depth are consistent with previous work [10,11,15], though Garcia-Sais *et al*. [13] found a small increase in abundance at 25m associated with a peak in species richness. These fine-scale changes in abundance are most likely driven by patterns in reef structural complexity, and so are locally driven. Unlike the shallow reef, most deep reef areas were not continuous reef, but large patch reef systems that the majority of fish were observed to aggregate around. This suggests that some of our abundance patterns could be driven by this changing reef structure with depth.

### Changes in fish trophic groups

Many of the fish trophic groups found on shallow reefs were also recorded on MCEs, with surprisingly little change in proportion of the community. While we did not directly measure environmental variables across the depth gradient, some of this consistency may result from similar environmental conditions. While factors such as reef slope angle and visibility varied by site, they were fairly consistent across the depth gradient. The only exception was reef slope angle on the south shore, which was similar for 5, 15 and 25m depth bands within sites, but the seabed became much flatter allowing patch reef systems to develop at 40m depth. In other locations larger shifts in trophic guilds have been observed with depth. For example planktivores increased from 16% of individual fish recorded at 30m to 31% at 40m in Puerto Rico [10], whereas they did not significantly change on Utila, making up 49% and 36% of fish recorded at 25m and 40m respectively. Most previous work has shown planktivores to increase as a proportion of the community with depth [11,15], while herbivores decline [7,15]. It is not clear why planktivorous fish were observed to decline with depth on Utila, but many previous studies used simple abundance measures to assess changes in fish community. It is possible that these increases in planktivore abundance as a proportion of the total community may actually reflect a decline in planktivore biomass as a proportion of the community. Many of these other studies crossed the shallow to upper mesophotic, and upper mesophotic to lower mesophotic boundaries, so an increase in planktivores may be associated with deeper fish communities than we observed. These greater depths may be associated with larger differences in environmental variables, such as temperature and luminosity affecting habitat types and driving species distributions. As previously mentioned, the 40m transects at the majority of our sites were close to the maximum bathymetric extent of reefs at the site. Therefore, this increase in planktivorous species could require a deeper reef extent than at our surveyed sites. In addition, smaller-bodied reef fish such as planktivores often require benthic habitat complexity for shelter [38]. We found lower hard coral and sponge cover and greater sand cover on MCEs than shallower reefs, suggesting that reduced habitat availability could play a role in planktivore decline. While the proportion of the community composed by planktivores at different depths is variable, many studies have recorded an overall decline in planktivorous fish abundance with depth [10,11] which agrees with our results on Utila.

Declines in herbivores shown here are more consistent with previous studies, with herbivores representing 36% and 26% of fish at 25m and 40m on Utila compared to 31% (30m) and 21% (40m) in Puerto Rico [10] and 40% (30m) and close to 0% (90m) in the Marshall Islands [11]. These patterns followed similar declines in macroalgal coverage with depth. While herbivorous reef fish occur at lower abundances (relative and proportional) on MCEs compared to shallow reefs they are still ecologically important. Lesser *et al*. [47] reported that following lionfish invasion of a Caribbean MCE there was a decline in herbivorous reef fish to 60m which led to a benthic phase shift to an algal-dominated mesophotic community. Reef fish, despite their lower abundance, are likely to be the dominant herbivores on MCEs, as the influence of *Diadema antillarum* urchins (the Caribbean’s primary herbivorous echinoderm) declines with depth [48]. For example in Curacao, following a mass *Diadema* mortality event in 1983, there was less change in algal coverage at 40m depth than at shallower sites [49,50]. This suggests that despite low herbivorous reef fish abundance in upper MCEs they have a crucial functional role in maintaining benthic reef health. Exact reasons for declines in herbivorous reef fish with depth are not clear, although there have been suggestions that changes in algal species, abundance or growth rates may be responsible [51–53]. Algal growth rates correlate with the availability of light which decreases along the depth gradient [54], and so a decline in algal productivity per unit area of substrate can be expected with depth.

Other suggested drivers of this bathymetric herbivore variation include differences in reef structure and changes in sedimentation rates. A study investigating algal recruitment and growth dynamics in the Caribbean using recruitment tiles found areas within close proximity (30-50m distance) to reefs at 30-35m depth had reduced algal abundance and herbivorous reef fish were observed grazing [54]. At greater depths algal abundance increased, and herbivorous fish declined except around artificial reefs (wrecks) at 50-60m [54]. Here, herbivory halos 10-20m wide surrounding the wrecks could be found with much lower algal abundance than nearby areas, suggesting that herbivorous reef fish were living within the wreck structure, implying reef structural complexity is important for deep-water herbivorous fish communities.

The two herbivorous acanthurid and two pomacentrid species interestingly did not significantly change in their proportion of the overall biomass-weighted fish community at shallow and MCE depths. This suggests that much of the decline in herbivores at mesophotic depths comes from a decline in scarids. Detailed diet studies conducted in the southern Caribbean suggest differences in diets, digestion and feeding of the acanthurid and scarid species encountered in our study might explain why scarids are lost more quickly with depth [55]. Scarid species generally feed on more detritus than acanthurids [55], although the Atlantic blue tang (*Acanthurus coeruleus*) also has a diet similar to many scarid species and does not decline with depth in our results. Previous work has identified rapid scarid declines across shallow depth gradients in the Caribbean [56], with declines in coral cover with depth impacting their supplementary feeding on coral polyps. For example, abundance of the Redband parrotfish (*Sparisoma aurofrenatum*), one of the scarids we observed declining with depth, is positively correlated with coverage of the boulder star coral (*Orbicella franksi*) [57].

Piscivores were observed to increase in relative biomass with depth in a similar pattern to other locations [11,15]. This reflects the shift away from herbivory in the community, as well as potential refuge effects. For example, studies have observed that large-bodied piscivorous species targeted by fisheries are often found in higher abundance at mesophotic depths [10], with depth acting as a refuge. Despite this increase in piscivores relative biomass on MCEs, it has been suggested that a large proportion of feeding carried out by large bodied piscivores and carnivores found on MCEs is actually conducted on shallow reefs [58]. This implies movement of individual large bodied piscivores and carnivores between the depth bands, and suggests these piscivores may therefore play an important role in transporting nutrients between shallow reefs and MCEs. Invertebrate feeder results are also broadly consistent on Utila with those found in Puerto Rico, with declines in abundance from 19% (25m) to 16% (40m) on Utila and 30% (30m) to 27% (40m) in Puerto Rico [10].

### Changes in fish lengths

Length distributions varied between shallow and mesophotic depths across the whole community. This can be explained by the high numbers of small-bodied fish at shallow depths, and is supported by the differences in the proportion of trophic groups based on abundance or biomass weighting, with the most abundant small fish being planktivores. Six species with ≥20 individuals recorded on shallow and deep reefs (blue tang, striped parrotfish, blue chromis, creole wrasse, bluehead wrasse, yellowtail snapper) exhibited increased body length with depth. In the Caribbean, previous work has identified ontogenetic migrations in the striped parrotfish and yellowtail snapper, with movement from shallower water to deeper reef habitats as juveniles mature to adults [23]. Finding larger body lengths of both these species at increased depth suggests that upper MCEs are incorporated into this ontogenetic migration.

There could be several explanations for this effect of depth on body size, which likely varies by species. For larger roaming reef fish species, such as the striped parrotfish and yellowtail snapper, larger individuals may be less susceptible to predation, and so are able to roam to greater depths where there is reduced structural complexity and thus they are more exposed. It has been shown experimentally that, in the absence of predators, fish occupy the habitat allowing the best growth rate, whereas when predators are present fish are associated with more structural complexity as a refuge, thus reducing vulnerability [59,60]. Work on Caribbean Nassau grouper (*Epinephelus striatus*) suggests that juveniles face a trade-off between maximising growth rates and minimising predation risk, and are associated with habitats that minimise the ratio of mortality risk to growth rate [22], while similar patterns have been observed for French grunt (*Haemulon flavolineatum*) [61]. However, increases in blue chromis and blue-headed wrasse lengths with depth are harder to explain as both are small-bodied planktivores. Blue-headed wrasse are known to act as cleaner fish, with cleaner activity declining with depth on the reef [62], suggesting increased reliance on planktivory at deeper sites. Blue chromis recruitment has also been observed to decline with depth across a 10-40m depth gradient, with no recruits recorded below 30m [63]. This suggests that larger individuals at greater depth are the result of individuals moving deeper.

## Conclusions

We investigated patterns in reef fish communities and fish trophic groups across shallow to mesophotic gradients on the southern Mesoamerican Barrier Reef, providing the most detailed study conducted in this region. We found reef fish species richness, abundance and biomass declined with increased depth. Importantly, we identify differences between fish community composition calculated based on abundance and those based on biomass. By using biomass, unlike many previous studies, we found no difference in planktivorous fish relative community composition across the depth gradient, while we detected declines in herbivores with increased depth and an increase in piscivores. This suggests future fish community comparisons across depth gradients should incorporate fish biomass assessments to provide better estimates of trophic composition.

## Supporting Information

S1 Appendix Description of data contained in S1, S2 and S3 Data. (DOC)

S1 Data Raw data used for the abundance and biomass based fish community ecology estimates. (CSV)

S2 Data Raw fish length data. (CSV)

S3 Data Raw benthic coverage data. (CSV)

S1 Table Coordinates of survey sites. All GPS points recorded in WGS84 and represent the centre of all transects conducted at the site. Site numbers indicate site location on Fig 1.
(DOCX)

S2 Table Species correlating with changes in their trophic group with depth. Results for species exhibiting a >0.3 absolute value correlation with the first Principle Components Analysis axis for their trophic group. Correlations were tested using a PERMANOVA based on biomass per transect, and the percentage of biomass contribution to total biomass of that guild per transect. Significant changes are indicated and the direction of change shown.
(DOCX)
