# Supplementary material for: Reef Fish Community Biomass and Trophic Structure Changes across Shallow to Upper-Mesophotic Reefs in the Mesoamerican Barrier Reef, Caribbean
Source: PLoS One. 2016 Jun 22;11(6):e0156641. doi: 10.1371/journal.pone.0156641 (PMC4917088; doi:10.1371/journal.pone.0156641)
Supplement: S1 Appendix — (DOC) [file pone.0156641.s001.doc]

**S1 Appendix**

**Description of data contained in Data Files S1, S2 and S3.**

All data files are provided as comma separated value (csv) files.

**S1 Data File**

This file contains the raw data used for the abundance and biomass based fish community ecology estimates. Each row represents a different individual fish. When conducting stereo-video surveys some fish were recorded on transects, but due to their body angle or closeness to the cameras it was not possible to accurately measure their body length. In this data file, any individual fish that we were unable to measure have had the mean body length for other individuals added following the procedure outlined in the methods section.

Summary of columns:

site – The survey site, Black Coral Wall (BCW), Coral View (COV), Little Bight (LBI), Lighthouse reef (LHO), Spotted Bay (SPB), Stingray Point (SRP) and The Maze (TMA). See Table 1 for full GPS locations of each site.

zone – The depth in meters (m) that the survey was conducted.

transect – The transect (replicate) number that the individual fish was recorded on.

name – Taxonomic name of the individual fish to the highest taxonomic resolution possible from the video. Recorded in the form: “Family_Genus_species”

family – Fish family name

genus – Fish genus name

species – Fish species name

length.mm – The measured or estimate length for the individual fish in millimetres (mm). See methods section and note above for details on estimated lengths.

weight.g – The estimated weight of the individual fish in grams (g).

**S2 Data File**

This data file contains the raw length data underpinning the S1 Data File. The S2 Data File was used for the fish length distribution analysis. Each row represents an individual unique fish recorded on a transect. All fish lengths in the S2 Data File are actual fish lengths measured using the stereo-video system. Fish that we were unable to accurately measure lengths for are included with a length of NA rather than being allocated a mean length.

Summary of columns:

Site – The survey site, Black Coral Wall (BCW), Coral View (COV), Little Bight (LBI), Lighthouse reef (LHO), Spotted Bay (SPB), Stingray Point (SRP) and The Maze (TMA). See Table 1 for full GPS locations of each site.

Zone – The depth in meters (m) that the survey was conducted.

Transect – The transect (replicate) number that the individual fish was recorded on.

Family – Fish family name

Genus – Fish genus name

Species – Fish species name

Length.mm – The measured length for the individual fish in millimetres (mm). See methods section and note above for details on estimated lengths.

**S3 Data File**

The S3 Data File contains the raw benthic coverage data collected by video point intercept transect. Each transect was 50m long, and point intercept analysis was conducted at 25cm intervals, generating 201 benthic observations per transect. Each row represents a unique benthic observation on a transect.

Summary of columns:

site – The survey site, Black Coral Wall (BCW), Coral View (COV), Little Bight (LBI), Lighthouse reef (LHO), Spotted Bay (SPB), Stingray Point (SRP) and The Maze (TMA). See Table 1 for full GPS locations of each site.

zone – The depth in meters (m) that the survey was conducted.

transect – The transect (replicate) number that the individual fish was recorded on.

point.intercept – The distance in meters (m) represented by the benthic observation along the transect tape. There are 201 observations per transect, at 25cm intervals starting at 0m and ending at 50m.

code – The benthic coverage category. Categories are adapted from English *et al.* (1997). Categories are: Anenome (AN), Coralline Algae (CA), Dead Coral (DC), Hard (Scleractinian) Coral (HC), Macroalgae (MA), Rock (RCK), Rubble (R), Sand (S), Soft Coral (SC), Sponge (SP), Turf Algae (TA), Tunicate (TU), Water (WA) and Zoanthid (ZO).

detailed – more detailed analysis of the benthic category, for example genus or species identification where possible and relevant.
