# Supplementary material for: Reef Fish Community Biomass and Trophic Structure Changes across Shallow to Upper-Mesophotic Reefs in the Mesoamerican Barrier Reef, Caribbean
Source: PLoS One. 2016 Jun 22;11(6):e0156641. doi: 10.1371/journal.pone.0156641 (PMC4917088; doi:10.1371/journal.pone.0156641)
Supplement: S1 Table — All GPS points recorded in WGS84 and represent the centre of all transects conducted at the site. Site numbers indicate site location on Fig 1. (DOCX) [file pone.0156641.s005.docx]

**S1 Table. GPS Coordinates of survey sites.**
All GPS points recorded in WGS84 and represent the centre of all transects conducted at the site. Site numbers indicate site location on Figure 1.

| Site Number | Site | Latitude | Longitude |
| --- | --- | --- | --- |
| 1 | Spotted Bay | 16.09713416 | -86.98717118 |
| 2 | The Maze | 16.11266214 | -86.94911793 |
| 3 | Stingray Point | 16.06890550 | -86.95477948 |
| 4 | Little Bight | 16.07926302 | -86.92942222 |
| 5 | Black Coral Wall | 16.08305968 | -86.91699554 |
| 6 | Coral View | 16.08823274 | -86.91094506 |
