# Supplementary material for: Reef Fish Community Biomass and Trophic Structure Changes across Shallow to Upper-Mesophotic Reefs in the Mesoamerican Barrier Reef, Caribbean
Source: PLoS One. 2016 Jun 22;11(6):e0156641. doi: 10.1371/journal.pone.0156641 (PMC4917088; doi:10.1371/journal.pone.0156641)
Supplement: S2 Table — Results for species exhibiting a >0.3 absolute value correlation with the first Principle Components Analysis axis for their trophic group. Correlations were tested using a PERMANOVA based on biomass per transect, and the percentage of biomass contribution to total biomass of that guild per transect. Significant changes are indicated and the direction of change shown. (DOCX) [file pone.0156641.s006.docx]

**S2 Table. Species correlating with changes in their trophic group with depth.** Results for species exhibiting a >0.3 absolute value correlation with the first Principle Components Analysis axis for their trophic group. Correlations were tested using a PERMANOVA based on biomass per transect, and the percentage of biomass contribution to total biomass of that guild per transect. Significant changes are indicated and the direction of change shown.

| Trophic Group | Species | Mean Biomass per transect/g ± SE | | | | | Mean Percentage of Trophic Group Biomass per transect/g ± SE | | | | |
| --- | --- | --- | --- | --- | --- | --- | --- | --- | --- | --- | --- |
|  |  | 5m | 40m | Pseudo F | p(perm) | Direction | 5m | 40m | Pseudo F | p(perm) | Direction |
| Herbivores |  |  |  |  |  |  |  |  |  |  |  |
|  | *Acanthurus bahianus* | 114.72 ± 51.32 | 7.42 ± 7.42 | 2.87 | 0.051 | - | 2.45 ± 1.12 | 1.1 ± 1.1 | 0.67 | 0.405 | - |
|  | *Acanthurus coeruleus* | 1265.46 ± 709.58 | 138.81 ± 77.29 | 1.66 | 0.178 | - | 20.2 ± 4.16 | 12.66 ± 6.1 | 1.12 | 0.306 | - |
|  | *Microspathodon chrysurus* | 42.39 ± 11.7 | 1.78 ± 1.55 | 7.93 | 0.002 | 🡻 | 1.76 ± 0.58 | 1.44 ± 1.36 | 0.06 | 0.874 | - |
|  | *Stegastes adustus* | 16.81 ± 3.02 | 0.84 ± 0.63 | 18.22 | <0.001 | 🡻 | 2.52 ± 1.29 | 3.58 ± 3.57 | 0.10 | 0.786 | - |
|  | *Scarus iserti* | 320.34 ± 68.8 | 107.73 ± 53.39 | 5.01 | 0.010 | 🡻 | 19.79 ± 3.85 | 16 ± 5.92 | 0.32 | 0.580 | - |
|  | *Sparisoma aurofrenatum* | 149.45 ± 63.62 | 0.6 ± 0.6 | 3.63 | 0.012 | 🡻 | 8.28 ± 2.95 | 0.09 ± 0.09 | 5.12 | 0.012 | 🡻 |
|  | *Sparisoma chrysopterum* | 110.39 ± 36.31 | 0 ± 0 | 6.13 | 0.006 | 🡻 | 2.7 ± 1.01 | 0 ± 0 | 4.77 | 0.011 | 🡻 |
|  | *Sparisoma rubripinne* | 178.39 ± 59.08 | 0 ± 0 | 6.05 | 0.009 | 🡻 | 5.95 ± 2.24 | 0 ± 0 | 4.67 | 0.023 | 🡻 |
|  | *Sparisoma viride* | 653.78 ± 121.76 | 15.14 ± 10.53 | 18.19 | <0.001 | 🡻 | 28.9 ± 4.69 | 5.63 ± 3.84 | 12.61 | 0.001 | 🡻 |
| Invertebrate Feeders |  |  |  |  |  |  |  |  |  |  |  |
|  | *Abudefduf saxatilis* | 2203.3 ± 872.39 | 0 ± 0 | 4.23 | 0.012 | 🡻 | 46.72 ± 6.6 | 0 ± 0 | 33.27 | <0.001 | 🡻 |
|  | *Lutjanus apodus* | 586.2 ± 303.81 | 0 ± 0 | 2.47 | 0.073 | - | 17.31 ± 5.42 | 0 ± 0 | 6.77 | 0.010 | 🡻 |
|  | *Lutjanus jocu* | 309.76 ± 296.39 | 20.02 ± 20.02 | 0.63 | 0.904 | - | 3.36 ± 2.55 | 3.57 ± 3.57 | 0.00 | 1.000 | - |
|  | *Lutjanus mahogoni* | 307.22 ± 168.65 | 0 ± 0 | 2.20 | 0.053 | - | 23.08 ± 5.99 | 0 ± 0 | 9.84 | 0.003 | 🡻 |
| Omnivores |  |  |  |  |  |  |  |  |  |  |  |
|  | *Kyphosus sectatrix* | 1322.45 ± 853.12 | 0 ± 0 | 1.59 | 0.280 | - | 9.52 ± 4.58 | 0 ± 0 | 2.86 | 0.142 | - |
| Piscivores |  |  |  |  |  |  |  |  |  |  |  |
|  | *Aulostomus maculatus* | 9.24 ± 5.45 | 0 ± 0 | 1.91 | 0.253 | - | 7.14 ± 4.02 | 0 ± 0 | 2.09 | 0.252 | - |
|  | *Caranx ruber* | 322.05 ± 219.45 | 75.47 ± 41.1 | 0.82 | 0.567 | - | 17.57 ± 5.7 | 17.86 ± 7.37 | 0.00 | 1.000 | - |
|  | *Sphyraena barracuda* | 77.9 ± 77.9 | 259.99 ± 205.87 | 0.89 | 0.471 | - | 2.38 ± 2.38 | 7.14 ± 4.96 | 0.91 | 0.568 | - |
| Planktivores |  |  |  |  |  |  |  |  |  |  |  |
|  | *Clepticus parrae* | 852.01 ± 717.26 | 4.67 ± 3.26 | 0.93 | 0.401 | - | 14.99 ± 4.37 | 0.78 ± 0.57 | 6.96 | 0.008 | 🡻 |
|  | *Chromis cyanea* | 796.74 ± 253.3 | 58.46 ± 31.19 | 5.60 | 0.001 | 🡻 | 56.03 ± 4.91 | 15.32 ± 6.43 | 25.99 | <0.001 | 🡻 |
